# Culture dependent and independent analyses suggest a low level of sharing of endospore-forming species between mothers and their children

**DOI:** 10.1038/s41598-020-58858-y

**Published:** 2020-02-04

**Authors:** Ekaterina Avershina, Marte Gro Larsen, Marina Aspholm, Toril Lindback, Ola Storrø, Torbjørn Øien, Roar Johnsen, Knut Rudi

**Affiliations:** 1 Department of Chemistry, Biotechnology and Food Science, Norwegian University of Life Sciences, 1433 Ås, Norway; 2 Faculty of Applied Ecology, Agricultural Sciences and Biotechnology, Inland Norway University of Applied Sciences, 2316 Hamar, Norway; 3 Department of Food Safety and Infection Biology, Faculty of Veterinary Medicine, Norwegian University of Life Sciences, 369 Sentrum, 0102 Oslo, Norway; 4 Department of Public Health and Nursing, Norwegian University of Science and Technology, 7491 Trondheim, Norway

**Keywords:** Bacteria, Microbiome

## Abstract

Spore forming bacteria comprise a large part of the human gut microbiota. However, study of the endospores in gut microbiota is limited due to difficulties of culturing and numerous unknown germination factors. In this study we propose a new method for culture-independent characterization of endospores in stool samples. We have enriched DNA of spore-forming bacterial species from stool samples of 40 mother-child pairs from a previously described mother-child cohort. The samples were exposed to a two-step purification process comprising ethanol and ethidium monoazide (EMA) treatment to first kill vegetative cells and to subsequently eliminate their DNA from the samples. The composition of the ethanol-EMA resistant DNA was characterized by 16S rRNA marker gene sequencing. Operational taxonomic units (OTUs) belonging to the Clostridia class (OTU1: *Romboutsia*, OTU5: Peptostreptococcaceae and OTU14: *Clostridium* senso stricto) and one belonging to the Bacillus class (OTU20: *Turicibacter*) were significantly more abundant in the samples from mothers and children after ethanol-EMA treatment than in those treated with ethanol only. No correlation was observed between ethanol-EMA resistant OTUs detected in children and in their mothers, which indicates that a low level of spore-forming species are shared between mothers and their children. Anaerobic ethanol-resistant bacteria were isolated from all mothers and all children over 1 year of age. Generally, in 70% of the ethanol-treated samples used for anaerobic culturing, 16S rRNA gene sequences of bacterial isolates corresponded to OTUs detected in these samples after EMA treatment. We report a new DNA-based method for the characterization of endospores in gut microbiota. Our method has high degree of correspondence to the culture-based method, although it requires further optimization. Our results also indicate a high turnover of endospores in the gut during the first two years of life, perhaps with a high environmental impact.

## Introduction

The human gut microbiota is an undeniably important part of the human body, crucial for proper functioning of multiple processes such as digestion^1^, immune system^2,3^, brain functioning^4^ and response to drugs^5^. There is no agreement on whether gut colonization is initiated pre- or postnatally^6^. However, the most active stage of the bacterial recruitment in the gut starts at the moment of birth and the gut microbiota composition fluctuates drastically during first weeks of life^7,8^. In case of natural birth, infant gut microbiota initially resembles the vaginal microbiota of the mother, whereas infants born by caesarean section are colonized by skin-associated bacteria^9,10^. The most pronounced difference between gut microbiotas of vaginally-born and cesarean-born children is the absence of *Bacteroides* in the latter^11^. After onset of breastfeeding, the infant is constantly supplied with breastmilk-associated bacteria^12,13^. The breastmilk-diet strictly guards the infant gut microbiota by promoting bifidobacteria and by supressing development of an adult-associated microbiota^14^. After breastfeeding cessation, the gut microbiota gradually develops towards an adult profile through acquisition of Clostridia and other adult-associated bacterial species^8,15^. In adulthood, the gut microbiota is resilient to perturbations and becomes as unique as a fingerprint^16,17^. However, it can still be altered to a certain degree, since members of one household tend to share more microbes with each other than with other people^18^.

Although the maternal microbiota play an important role in shaping the infant gut microbiota^19^, newborns also seem to share as many bacteria with other women who have recently given birth as with their biological mothers^14^. Moreover, the part of the maternal microbiota that is highly prevalent in the population tend to be detected earlier in the newborn gut than the part that is detected in fewer individuals^14^. This suggests that even at early stages of life, children acquire bacteria from the environment in addition to those transmitted directly from their mothers.

An endospore is a highly stress resistant and non-reproductive differentiation state of bacteria that facilitate bacterial survival under conditions that are not suitable for growth. Germination of endospores, i.e. the process of re-entering the proliferative state, is initiated when the spores sense the presence of nutrient molecules (germinants), which indicate favorable growth conditions^20^. Up to 60% of the microbes in an adult gut are predicted to be capable of forming endospores^21^ and Clostridia comprise one of the most abundant bacterial classes in the gut^22^. Since Clostridia are strict anaerobes, it is still not clear how they colonize the gut, but they are most likely transmitted to humans as endospores. However, most studies on Clostridial spore germination are based on a few model species^23^, and there are therefore still large knowledge-gaps with regards to understanding the mechanisms for Clostridial colonization of the gut. Furthermore, a large fraction of spore-forming species in the gut microbiota are most probably not yet identified due difficulties to culture them and to extract DNA from spores.

In 2018, Kearney *et al*. suggested a culture-free method for the enrichment of DNA from endospores and other resistant cells based on the series of lysis treatments^24^. The present study describes an alternative culture-independent approach for characterizing the spore DNA by inactivating the DNA from the vegetative fraction of the bacterial community while keeping the spore DNA intact. In 2016, Browne *et al*. described spore forming bacteria from human stool by isolating the ethanol-resistant fraction of the stool samples followed by addition of a bile acid mixture to induce spore germination^21^. We have developed a two-step process for culture-independent spore characterization. During the first step, vegetative bacterial cells are weakened or killed by incubation in ethanol as previously described by Browne *et al*.^21^. Although ethanol treatment kills vegetative cells, it does not affect the DNA and therefore we employ the second step designed specifically to destroy DNA from the damaged cells and prevent its further amplification. At this step, the samples are mixed with the DNA intercalating agent ethidium monoazide (EMA) which is capable of penetrating the compromised cell wall of the vegetative cells, whereas the spores’ cell wall remains impermeable. Subsequent light treatment of the samples destroys the EMA and simultaneously shears the EMA intercalated DNA^25^. As a result, genomic DNA can only be isolated, amplified and sequenced from cells with non-compromised cell walls i.e. the ethanol-resistant fraction of the bacterial community. After treatment with ethanol and EMA we analyzed the taxonomic composition of the samples by 16S rRNA gene qPCR quantification and sequencing. The ethanol treated samples were also cultured to explore the composition of the culturable part of the fecal microbiota from mothers and their children.

This new culture-independent approach for enrichment of DNA from spores was employed to examine the ethanol resistant fraction of stool samples from forty mothers and their offspring, sampled throughout the two first years of the children’s life. Furthermore, 158 bacterial isolates were collected from the ethanol-resistant fraction of stool samples from eight randomly selected mother-child pairs. Taxonomical comparison of these isolates showed a high degree of overlap between the new culture-independent approach and a traditional culturing method for assessing ethanol-resistant fraction of the microbiota.

## Methods

**Figure 1:**
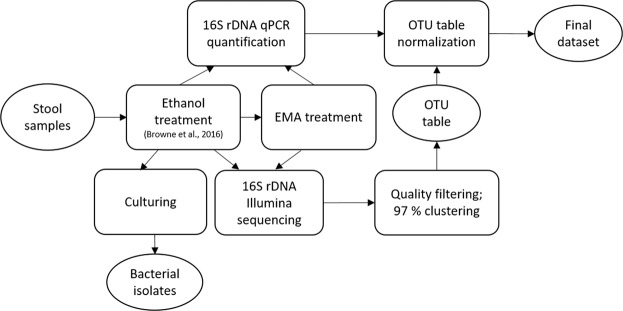
Schematic overflow of the study. Datasets are represented in oval and processes in rectangular shapes.

Schematic overview of the study workflow is shown in Fig. 1.

### Study cohort

In total, forty mother-child pairs were included in the present study. The mother-child pairs were selected from the IMPACT study^26^, based on the completeness of samples during the study period and on the expected level of spore-forming bacteria in the mothers^20^. For each mother-child pair, a total of five stool samples were analyzed using Illumina sequencing: one stool sample taken from the mother during pregnancy and four samples taken from the child: at 3 or 10 days, at 4 months, at 1 year and at 2 years of age. Further, eight of the forty mother-child pairs were randomly selected for culturing and characterization of ethanol resistant fraction of the microbiota. Four samples per mother-child pair were cultured; one from the mother and three from the child.

The Regional Committee for Medical Research Ethics for Central Norway approved the study (Ref. 120–2000). The study was granted a license by the Norwegian Data Inspectorate to process personal health data and one of the parents of each child signed a written informed consent form (Ref. 2003/953-3 KBE/-). Current controlled trials registration number: ISRCTN28090297. All experiments were performed in accordance with relevant guidelines and regulations.

### Inactivation of vegetative bacterial cells

Within the IMPACT study, the stool samples (2 g) were diluted in 10 ml of Cary-Blair medium, homogenized, aliquoted and stored at −80 °C. For this work, the 1 ml aliquots of the stool samples were thawed on ice, pulse centrifuged and 700 µl of the supernatants were diluted in a 1% NaCl solution (0.2 µm filter-sterilized). Stool samples from the mothers and the 1- and 2- year old children were diluted 9 times whereas stool samples from infants were diluted twice due to lower concentration of solid particles that may interfere with light inactivation. The samples were then incubated in freshly prepared 70% ethanol (1:1 ratio) at room temperature for 4 hours as previously described^21^. Subsequently, the samples were centrifuged for 5 min and the pellets were dissolved in 600 µl of 1% NaCl solution. Aliquots of the ethanol-treated samples were stored at −40 °C.

### Culturing of ethanol resistant bacteria

The ethanol-treated samples were thawed on ice and diluted 10 times in 1% NaCl solution (0.2 µm filter-sterilized) before 50 µL of the bacterial suspensions were streaked on anaerobically pre-conditioned YCFA medium agar plates substituted with 0.1% sodium taurocholate (Sigma-Aldrich)^21^ and on nutrient agar (Supplementary Text 1) for aerobic culturing. Culturing was performed at 37 °C for 24 hours both aerobically and anaerobically. After purification, the isolates were cultured for another 24 hours on the same medium, harvested and preserved for DNA extraction in 200 µL of STAR buffer (Roche). The DNA was extracted from the isolates using LGC MagMidi DNA extraction kit (LGC Genomics, UK) following the manufacturer’s recommendations. Nearly the full-length 16S rRNA gene (~1100 bp) was then amplified using GA-map^TM^ CoverAll primer pair (Genetic Analysis AS, Norway) and sent to GATC Biotech (GATC Biotech, Germany) for Sanger sequencing.

### Fragmentation of DNA from vegetative bacterial cells by EMA

Ethidium monoazide (EMA; Thermo-Scientific) is a DNA intercalating agent that was added to the ethanol-treated stool samples to a final concentration of 2.5 ng/µL (these samples are later on referred to as ethanol-EMA treated samples). Photoactivated EMA directly cleaves bacterial DNA^25^ and after five minutes of incubation in dark, the samples were exposed to light in a photo activation universal light (PAUL) platform (GenIUL, Spain) for 30 min.

### DNA extraction and PCR amplification

DNA was extracted both after ethanol treatment and after ethanol-EMA treatment. The stool samples were first mechanically lysed with 0.25 g of acid-washed glass beads (<106 µm, Sigma-Aldrich) using 2 rounds of 20 seconds bead-beating with 1-min rest in between. Finally, the homogenized samples were subjected to automated magnetic bead-based DNA extraction on KingFisher Flex system (Thermo Scientific) using LGC MagMidi kit (LGC Genomics, UK) by following the manufacturer’s recommendations. The V3 - V4 region of 16S rRNA gene was amplified using Illumina-modified 16S rRNA gene universal PRK primers (PRK341F: 5′-CCTACGGGRBGCASCAG-3′ and PRK806R: 5′-GGACTACYVGGGTATCTAAT-3′)^27^ and paired-end sequenced on MiSeq platform using v3 chemistry (Illumina). DNA extraction was performed on three 96-well plates and a negative control (PCR-graded water) was added to each run and subsequently sequenced. The total genomic DNA was quantified by real-time qPCR with PRK primers^27^ using a LightCycler LC480 platform (Roche) using DNA polymerase activation at 95 °C for 15 min followed by 40 cycles of 95 °C for 30 sec, 55 °C for 30 sec and 72 °C for 45 sec.

### Data analysis

Raw sequencing reads were joined using *fastq-join* algorithm implemented in *usearch* v 8.0^28^, demultiplexed, quality filtered (maxee <1) and clustered at 97% similarity level using *uparse* algorithm also implemented in *usearch* v 8.0^28^. Illumina barcodes and PRK primers were removed from sequences prior to clustering. In addition to *de novo* chimera detection, 97% operational taxonomic units (OTUs) were filtered against the ChimeraSlayer (“gold”) data base^29^. The taxonomy was assigned using the Greengenes v13.8 database^30^. Samples that had low 16S rDNA copy number counts after the ethanol treatment (ct > 30), were removed from the study. To reduce sequencing noise further, we excluded OTUs that represented less than 1% of the total bacterial load in the ethanol-treated fraction. Alpha-diversity was assessed using reciprocal Simpson’s index.

### Statistics

Kruskal-Wallis non-parametric test was used to assess significant differences between various groups and the resulting p-values were corrected for multiple testing using Benjamini-Hochberg correction. Furthermore, to assess whether the enrichment of the OTUs in maternal samples after the EMA treatment could be explained by chance, the ethanol-treated dataset was randomly subsampled by keeping the subsampling depth equivalent to the sequencing depth of the EMA-treated fraction. We then compared whether the number of 16S rRNA gene copies belonging to the significantly altered OTUs after the EMA treatment differed from the simulated ones.

Correlation in detection of OTUs between mother and child samples was assessed using Chi-square test. All statistical analyses were performed in MATLAB R2016b (MathWorks Ltd., USA).

FDR-corrected p-value (FDR p) of 0.05 was used as a cutoff for the significance.

### Ethics approval

This study is based on the samples obtained within the previosly published IMPACT study^26^. The Regional Committee for Medical Research Ethics for Central Norway approved the IMPACT study (Ref. 120–2000). The IMPACT was granted a license by the Norwegian Data Inspectorate to process personal health data and one of the parents of each child signed a written informed consent form (Ref. 2003/953-3 KBE/-). Current controlled trials registration number: ISRCTN28090297.

## Results

### General description of the dataset generated by 16S rRNA sequencing

The sequencing dataset comprised 4 267 716 accepted paired-end joined reads. The operational taxonomic unit (OTU) table counts were transformed by multiplying the proportion of reads belonging to a given OTU by the absolute 16S rRNA copy number estimated by qPCR. The average number of normalized 16S rRNA gene copies per samples was 315 340 ± 479 930 [mean ± standard deviation] and 7 030 ± 28 578 for ethanol and ethanol-EMA treated samples respectively. In total, our final dataset comprised data of 135 OTUs from 370 ethanol and EMA treated stool samples from 40 mother-child pairs.

**Figure 2:**
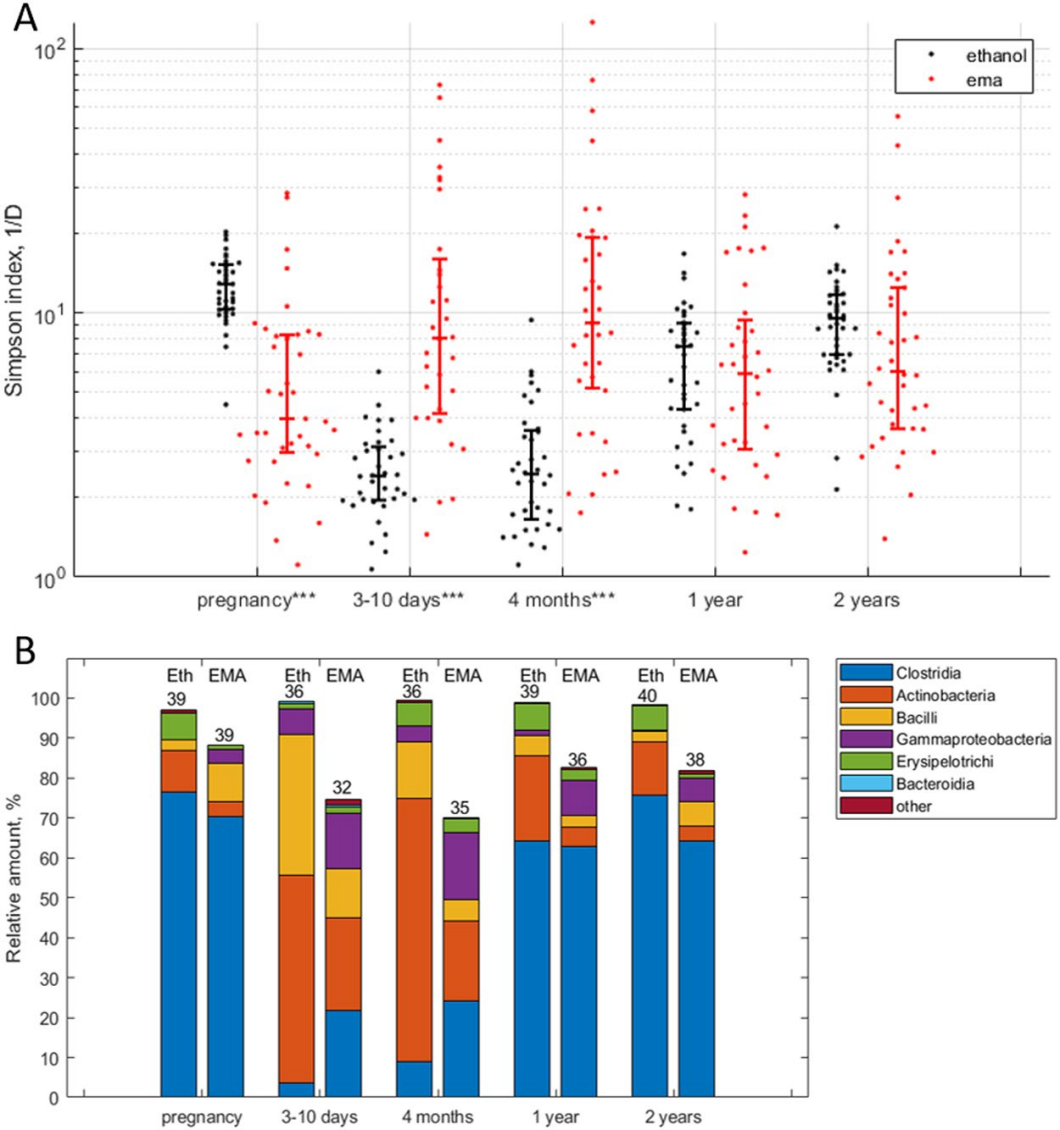
(**A**) Simpson’s reciprocal index of diversity after ethanol and ethanol-EMA treatment. *FDR < 0.05; **FDR < 0.01; ***FDR < 0.001. (**B)** Average bacterial class distribution of stool samples after ethanol and ethanol-EMA treatments, numbers above bars represent number of samples in each time and treatment category. Eth – Ethanol.

Reciprocal Simpson’s diversity index in samples from the mothers decreased from 12.9 (4.9) [median (IQR)] after ethanol treatment to 4.0 (5.3) (FDR p = 7.0·10^−9^) after ethanol-EMA treatment. We observed the opposite trend in stool samples from infants, where the EMA treated samples showed an increased Simpson’s diversity index from 2.4 (1.1) to 8.0 (11.8) and from 2.4 (1.9) to 9.2 (14.1) at 3–10 days and 4 months of age respectively (FDR p < 0.001; Supplementary Table 1) (Fig. 2A).

### Clostridia and Bacilli OTUs were enriched in stool samples after the ethanol-EMA treatment

Clostridia, Actinobacteria and Bacilli represented the three most abundant bacterial classes and collectively comprised around 90% of the total microbial load in ethanol-treated samples (Fig. 2B). Among these classes, Actinobacteria were reduced after ethanol-EMA treatment both in mothers and their children at all sampling points with the highest drop from 70.8% (33.8%) [median (IQR)] to 8.2% (15.2%) at 4 months of age (FDR p < 0.05; Supplementary Table 2). The Clostridia class was significantly enriched after ethanol-EMA treatment of stool samples from infants (0.6% (2.7%) to 11.3% (23.8%) and 2.7% (5.6%) to 15.8% (19.3%) at 3–10 days and 4 months of age, respectively; FDR p < 0.001; Supplementary Table 2), but not in the samples from mothers or from the 1 and 2 year old children. Bacilli was enriched from 1.4% (2.1%) after ethanol treatment to 5.1% (9.6%) after ethanol-EMA treatment (FDR p = 0.003) in the maternal samples. In contrast to the maternal samples, Bacilli were reduced in the samples from infants of 3–10 days and 4 months of age after ethanol-EMA treatment (25.3% (46.6%) to 6.9% (9.0%) and 11.9% (13.3%) to 3.8% (3.9%) respectively; FDR p < 0.05; Supplementary Table 2), whereas no change was detected in stool samples from 1 and 2 year old children.

**Figure 3:**
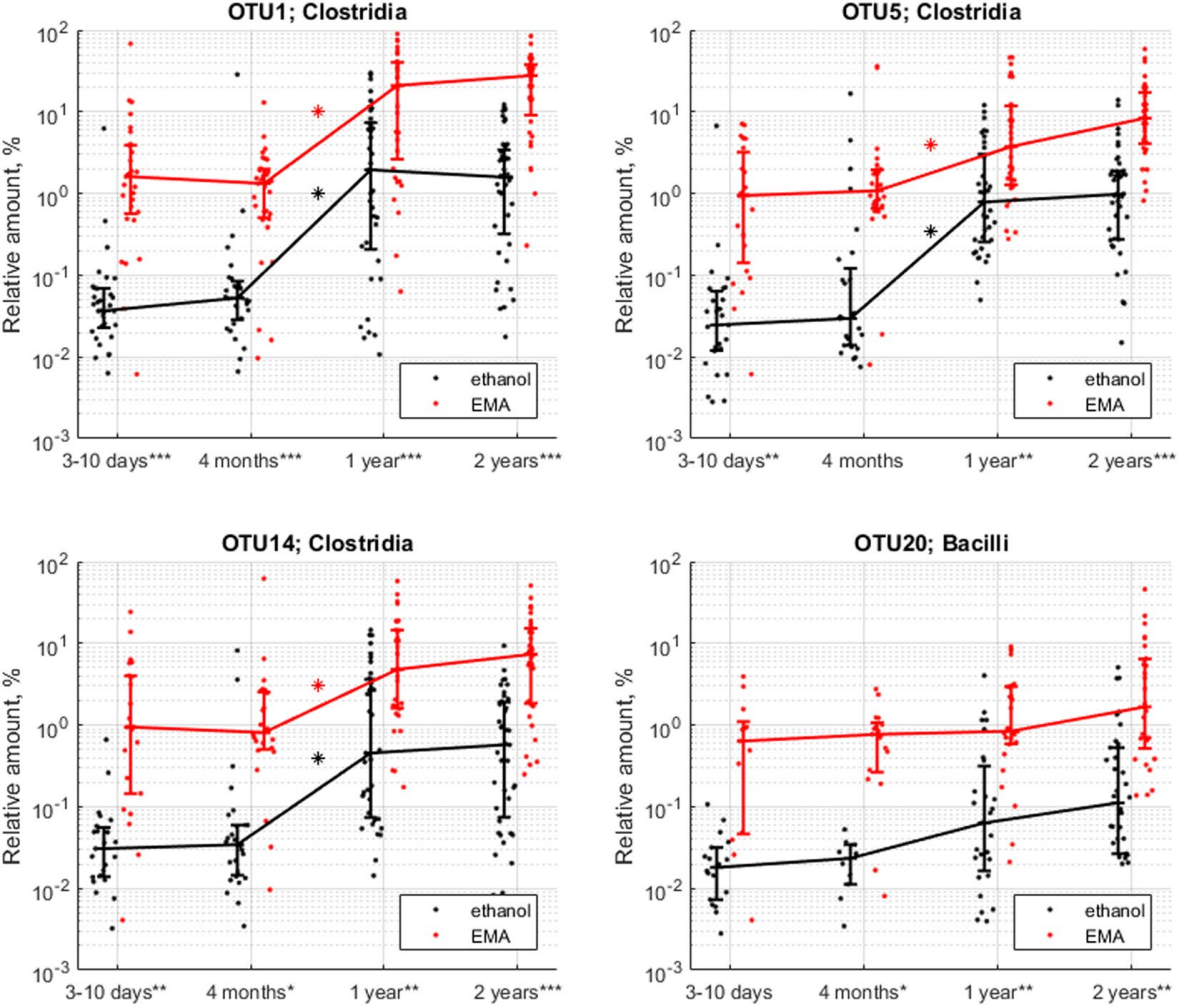
Relative amount of OTUs enriched in stool samples from children after ethanol-EMA treatment. *FDR < 0.05; **FDR < 0.01; ***FDR < 0.001.

In addition to the three most abundant bacterial classes, the Gammaproteobacteria class was also enriched after the EMA treatment in both mothers and children throughout the study (FDR p < 0.05; Supplementary Table 2) (Fig. 2), whereas Bacteroidia class was nearly absent at all time points regardless of the treatment. There was only one sample from a 10 days old child where the Bacteroidia class reached 20% of the relative microbial abundance after ethanol treatment. In the rest of samples this class never exceeded 2%. In samples from mothers, three OTUs belonging to the Clostridia class (OTU1: *Romboutsia*, OTU5: Peptostreptococcaceae and OTU14: *Clostridium* senso stricto) and one belonging to the Bacillus class (OTU20: *Turicibacter*), were significantly more abundant after ethanol-EMA treatment compared to ethanol treatment (FDR p < 0.05; Supplementary Table 3) (Supplementary Fig. 1). The same pattern was observed in samples from infants from they were 10 days old throughout the course of the study (FDR p < 0.05; Supplementary Table 4) (Fig. 3). The relative abundance of OTU1, OTU5 and OTU14 increased at 1 year of age regardless of EMA treatment, and then remained stable to the age of 2 years (FDR p < 0.05; Supplementary Table 5) (Fig. 3). OTU20, on the other hand, remained stable throughout the time course of the study (Fig. 3).

### Detection of EMA-stable OTUs in children is independent of their mothers

The ethanol-EMA enriched OTUs detected 10 days after birth remained persistently observed at subsequent sampling time points in the majority of the samples from children (Supplementary Table 6). To address the likelihood of mother-child transmission of EMA stable OTUs, we looked for co-occurrence of these OTUs in samples from mothers and their children by analyzing each time point independently. These comparisons showed no correlation between the occurrence of these OTUs between mothers and their children.

### Culturing of ethanol-resistant bacteria

Anaerobic ethanol-resistant bacteria were isolated from all mothers and all children after 1 year of age. Three out of four stool samples from 3-days-old children, one out of four samples from 10-days-old children, and two out of four samples from 4 months-old children contained culturable ethanol-resistant anaerobic bacteria (Supplementary Table 7).

DNA was extracted from all isolates and subjected to full 16S rRNA gene Sanger sequencing. In total, 158 bacterial isolates had sequencing reads longer than 100 bp (Supplementary Table 8). One of three DNA extraction negative controls also contained detectable DNA identified as *Tatumella* species (Enterobacteriaceae) by 16S rRNA gene sequencing. The majority of the anaerobic isolates belonged to the Clostridia class with the exception of six isolates identified as *Turicibacter* (Erysipelotrichia). Bacterial cultures isolated aerobically, were identified as *Bacillus thuringiensis* (n = 3) and *Bacillus circulans* (n = 4).

The anaerobic isolates were assigned to 31 bacterial species based on their 16S rRNA gene sequences. Twenty-six of these species showed more than 97% similarity towards OTU sequences obtained by Illumina sequencing, and 21 of them were detected after ethanol-EMA treatment. Generally, bacterial isolates from 85% of the ethanol-treated samples used for anaerobic culturing, corresponded to OTUs detected in these samples by Illumina sequencing. In 59% of cases, these OTUs were also observed in the samples after they were treated with EMA. Cultured isolates from ethanol-treated samples belonging to OTU1, OTU5, OTU14 and OTU20 were also detected in the corresponding samples after the EMA treatment (Table 1). All isolates within the OTU1 group were identified as *Clostriduim dakarense* and within OTU20 – as *Turicibacter sanguinis*. Isolates with highest similarity towards OTU14 and OTU5 had heterogenous species assignments including *Intestinibacter bartlettii*, *Paeniclostridium sordelii*, *Clostridium celatum*, *Clostridium disporicum* and *Clostridium sartagoforme* (Supplementary Table 9).

**Table 1:** Relative amount of the EMA-enriched OTUs in the samples used for ethanol-resistant bacteria isolation.

ID	category	OTU_1	OTU_14	OTU_5	OTU_20
S1	mother - pregnancy	**28.02**	**26.86**	17.69	10.59
	child - 1 year	91.12	1.76	**1.24**	0.10
S2	child - 2 years	1.04	0.26	**61.14**	1.30
S3	mother - pregnancy	25.77	**15.08**	10.02	**9.76**
	child - 4 months	2.30	0.00	**51.72**	0.00
	child - 2 years	**24.33**	13.76	3.15	**0.38**
S4	child - 3 days	**1.87**	0.08	0.31	0.05
S5	mother - pregnancy	79.43	**0.55**	6.82	1.46
S6	mother - pregnancy	15.95	**6.90**	21.12	24.57
	child - 3 days	0.69	**13.79**	0.23	0.00
S7	mother - pregnancy	36.95	**2.75**	41.24	4.71
S8	child - 1 year	6.85	2.05	**8.22**	2.74

Samples, where the given OTU was successfully isolated, are highlighted in bold.

### Ethanol-resistant bacterial isolates shared between mother and child

Three out of eight mother-child pairs shared isolates of the same species. *Turicibacter sanguinis*, a genus in the Bacilli class that is often found in the intestine of animals^31^, was shared between a mother and her two-year-old child. *Sellimonas instestinalis*, a Gram-positive obligately anaerobic species representing a distinct phyletic line within the family Lachnospiraceae, was shared between a mother and her 4-month-old child and *Clostridium disporicum* was shared between a mother and her 3-days-old child. The two latter species showed 99.7% identity over the shared sequenced region of the 16S rRNA gene, whereas *Turicibacter sanguinis* isolates were 98.8% identical between mother and her child (Supplementary Table 10).

## Discussion

After cessation of breastfeeding, bacteria belonging to the class Clostridia increase to become major constituents of the human gut microbiome^8^. Since Clostridia are strictly anaerobic, they are likely to enter the human gut in the endospore form. We still don’t know if strictly anaerobic spore-forming species colonize the human gut immediately after the spores have entered the host or if the spores remain dormant in the intestine for a longer period until conditions are suitable for growth. Furthermore, it is not known if they are transmitted from the mother or if they come from the environment. In this work we have addressed these knowledge gaps by using a novel method for enrichment of DNA from the ethanol-resistant fractions of microbial populations, most likely representing spores, combined with 16S rRNA sequence analysis. We have examined stool samples from a subset of 40 mother-child pairs from a previously described mother-child cohort^26^.

It has previously been shown that ethanol treatment is effective for elimination of vegetative cells before recovery of bacterial spores from complex biological samples^21,32^. In this study we observed that Actinobacteria was the dominant phyla in the fecal samples of newborn and four months-old children but their abundance was reduced later in life. Unlike^24^, we observed reduction in Actinobacteria after the EMA treatment. Since many species belonging to the phylum Actinobacteria do not form spores, the observed reduction in abundance of this phylum after EMA treatment was therefore expected. The discrepancy between two studies might be explained by the differences in vegetative cells treatment. In^24^, authors use lysozyme for the enrichment of the resistant cells and Actinobacteria are resilient towards this enzyme. In our method, on the other hand, we rely on ethanol disruption of cell wall followed by light-activated shearing of DNA strands by EMA.

The increase of bacteria belonging to Gammaproteobacteria, observed in all samples after the EMA treatment, was unexpected. Gammaproteobacteria is a large class of Gram-negative species consisting of many harmless intestinal symbionts as well as familiar pathogens such as *Salmonella*, *Shigella* and *Yersinia*, which are not reported to be capable of forming spores. Since Gammaproteobacteria are common PCR contaminants^33^, their increase might indicate low abundance of spore DNA in these samples. Another surprising aspect was the very low detection of the Bacteroidia class in ethanol treated samples. Since Bacteroidetes DNA can be significantly affected by the bead-beating^34^, we believe that this might be a result of the harsh conditions the cells were subjected to.

Generally, infants have only few bacterial species in their gut at birth, and the diversity is gradually increasing throughout the first years of life^7,14,35^. This trend was also observed in the ethanol treated samples in the present study. However, the alpha-diversity in stool samples from newborns and 4-month-old children after ethanol-EMA treatment was higher than in the ethanol treated samples, whereas in the mother samples ethanol-EMA treatment significantly reduced alpha diversity. In this work, we used Simpson’s index as the measure of alpha-diversity. This index takes both number of species (richness) and their fractions (evenness) into account. Therefore, higher alpha-diversity may indicate that after the EMA treatment, although number of detected OTUs drops, they become more evenly distributed resulting in the higher Simpson’s index. By the age of one year, alpha diversity of ethanol-EMA treated samples was comparable to that of the ethanol treated samples, indicating a higher domination of endospore-forming bacteria.

Three Clostridia and one Bacilli OTU were significantly overrepresented in the ethanol-EMA-resistant fractions corroborating previous observations^24^. The Clostridial OTUs remained on the same level during the first 4 months of life and then significantly increased by the age of one, whereas Bacilli OTU remained stable throughout the whole study period.

Isolates belonging to ethanol-EMA enriched Bacilli OTU were identified as *Turicibacter sanguinis*. *Turicibacter saguinis* was initially reported as a non-spore-forming bacteria^31,36^, however, it has later been shown that it is able to form endospores^21^. Clostridia ethanol-EMA enriched OTUs comprised species of *Clostridium dakarense*, *Intestinibacter bartlettii*, *Paeniclostridium sordellii*, *Clostridium celatum*, *Clostridium disporicum* and *Clostridium sartagoforme*. All of these species were demonstrated to have spore-forming ability^37–43^. *Clostridium dakarense* was first described in 2013 after having been isolated from a 4-month-old Senegalese infant with gastroenteritis^43^. *Intestinibacter bartlettii*, previously known as *Clostridium bartlettii*^44^, was previously reported to be widespread among the healthy adult Japanese population^37^. *Paeniclostridum sordellii* strains are mostly non-pathogenic, although some strains cause severe non-gastrointestinal infections^45^.

Many studies have demonstrated vertical transmission of bacterial strains between mothers and their children^46–48^. Contrastingly, in the present work both 16S rRNA sequencing and culturing based experiments indicate low levels of sharing of ethanol-EMA-resistant OTUs between mothers and their children. At the same time, we observed stable colonization of children gut by ethanol-EMA stable bacteria suggesting environmental acquisition of these bacteria. It is important to note though, that in our work we only address transmission of spores, but not of vegetative cells of spore-forming bacteria.

Ethanol treated stool samples were selected from eight mother-child pairs and used for aerobic and anaerobic culturing. Aerobic ethanol-resistant bacteria were only detected in two samples; whereas anaerobic bacteria were detected in the majority of samples. Stool samples from mothers and one- and two-year-old children were rich in anaerobic ethanol-resistant bacteria. Interestingly, the rate of detection of these bacteria in stool samples of 3-day-old infants was higher than in 10-day-old infants, possibly indicating a high turnover of these bacteria. The culturing experiments also showed a low level of sharing of spore-forming bacterial between children and their mothers.

Using the approach described in this study, we classified only four ethanol-EMA resistant OTUs. Since all of these OTUs also were isolated by culturing from the corresponding samples, we suggest that EMA treatment retain spore DNA from the ethanol-resistant fraction of bacteria. Many of the isolates from culturing the ethanol-resistant fraction of the stool samples were detected in the corresponding 16S rDNA dataset, but not classified as ethanol-EMA resistant. We have applied very stringent conditions for the classification of the EMA resistant OTUs to prevent false positive errors and therefore the method requires further optimization for labelling OTUs as ethanol-EMA resistant.

## Conclusion

In conclusion, we report a new method for culture-independent assessment of bacterial spores from complex microbial communities. By using this method, we found evidence for a low level of maternal transmission of spore forming bacteria suggesting that they are mainly acquired from environment. The sources of acquisition remain an interesting subject for further studies.

## Supplementary information

Supplementary Material.

## Data Availability

The datasets used and analysed during the current study are available from the corresponding author on reasonable request.
