# Supplementary material for: Culture dependent and independent analyses suggest a low level of sharing of endospore-forming species between mothers and their children
Source: Sci Rep. 2020 Feb 4;10:1832. doi: 10.1038/s41598-020-58858-y (PMC7000398; doi:10.1038/s41598-020-58858-y)
Supplement: Supplementary file 1 — Supplementary Material. [file 41598_2020_58858_MOESM1_ESM.docx]

**Supplementary Material**

Culture dependent and independent analyses suggest a low level of sharing of endospore-forming species between mothers and their children

Ekaterina Avershina^1,2*^, Marte Gro Larsen^1^, Marina Aspholm^3^, Toril Lindback^3^, Ola Storrø^4^, Torbjørn Øien^4^, Roar Johnsen^4^ and Knut Rudi^1^

^1^Department of Chemistry, Biotechnology and Food Science, Norwegian University of Life Sciences, 1433 Ås, Norway

^2^Faculty of Applied Ecology, Agricultural Sciences and Biotechnology, Inland Norway University of Applied Sciences, 2316 Hamar, Norway

^3^Department of Food Safety and Infection Biology, Faculty of Veterinary Medicine, Norwegian University of Life Sciences, 369 Sentrum, 0102 Oslo, Norway

^4^Department of Public Health and Nursing, Norwegian University of Science and Technology, 7491 Trondheim, Norway

*corresponding author: [ekaterina.avershina@gmail](mailto:ekaterina.avershina@gmail).com; +47 6251 7753

**Supplementary Text 1**. Nutrient agar composition

| Beef Extract | 3.0 g |
| --- | --- |
| Peptone | 5.0 g |
| Agar | 15.0 g |
| Distilled Water | 1000 ml |

Final pH 6.8 +/- 0.2.

**Supplementary Figure 1.** Detection of ethanol-EMA enriched OTUs. True number of 16S rRNA copies of OTUs in stool samples after ethanol-EMA treatment as compared to random number of 16S rRNA gene copies belonging to these OTUs given the depth of ethanol sample sequencing as subsampling depth


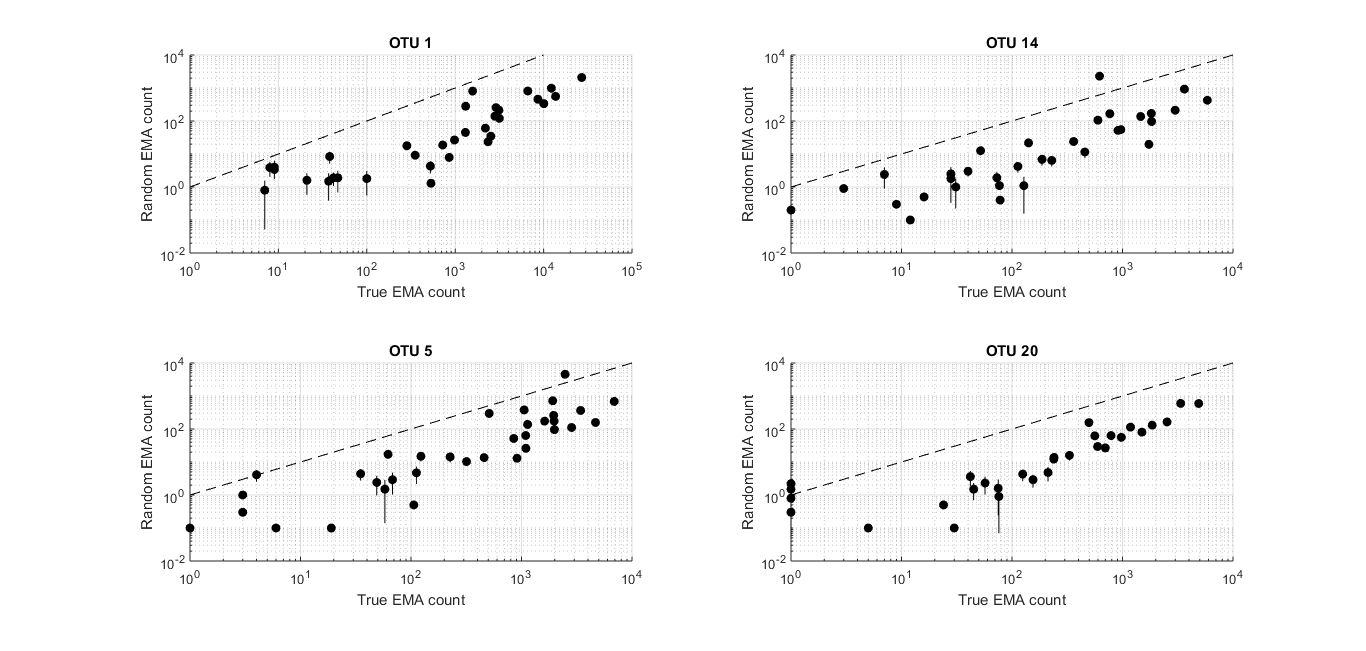


**Supplementary Table** **1**. FDR-corrected p-values of differences between Simpson's Reciprocal index of ethanol- and ethanol+EMA

|  | pregnancy | 3-10days | 3 months | 1 year | 2 years |
| --- | --- | --- | --- | --- | --- |
| FDR p-value | 3.50E-08 | 5.27E-08 | 7.80E-08 | 0.464335 | 0.053032 |

**Supplementary Table 2**. FDR-corrected p-values of differences between relative abundance of bacterial classes between ethanol- and ethanol+EMA treated samples

| Time point | Clostridia | Actinobacteria | Bacilli | Gammaproteobacteria | Erysipelotrichia | Bacteroidia | Coriobacteriia | Verrucomicrobiae | Chloroplast | Methanobacteria | Unassigned |
| --- | --- | --- | --- | --- | --- | --- | --- | --- | --- | --- | --- |
| mothers - pregnancy | 6.86E-01 | 2.24E-06 | 2.83E-03 | 7.55E-09 | 3.07E-10 | 1.21E-03 | 5.13E-10 | 2.83E-03 | 2.63E-01 | 1.80E-01 | 4.15E-01 |
| 3-10 days | 4.37E-07 | 2.53E-02 | 5.95E-03 | 2.53E-02 | 2.82E-01 | 7.82E-01 | 2.82E-01 | 2.82E-01 | 3.32E-01 | 3.80E-01 | 3.22E-01 |
| 4months | 1.35E-05 | 5.65E-06 | 1.03E-03 | 1.85E-05 | 3.48E-01 | 6.75E-01 | 5.11E-01 | 9.00E-01 | 2.97E-01 |  | 4.48E-01 |
| 1year | 7.91E-01 | 1.50E-06 | 2.26E-01 | 1.77E-05 | 1.27E-04 | 1.28E-02 | 1.28E-02 | 4.67E-04 | 3.09E-01 | 1.44E-01 | 5.41E-01 |
| 2years | 3.21E-01 | 2.21E-07 | 1.67E-01 | 3.77E-08 | 6.51E-09 | 6.83E-04 | 1.28E-04 | 2.85E-05 | 1.11E-01 | 9.56E-01 | 9.56E-01 |

| **Supplementary Table** **3.** FDR-corrected p-value of differences between true and randomly generated number of reads belonging to a given OTU in a ethanol-EMA treated dataset   \| OTU_num \| OTU_1 \| OTU_14 \| OTU_5 \| OTU_20 \| \| --- \| --- \| --- \| --- \| --- \| \| FDR p \| 2.53E-05 \| 4.78E-05 \| 0.000293 \| 0.000315 \|   **Supplementary Table** **4.** FDR-corrected p-value of differences in relative abundance of a given OTU between ethanol- and ethanol+EMA   \| Ethanol vs EMA; BH-FDR \| 3-10days \| 3 months \| 1 year \| 2 years \| \| --- \| --- \| --- \| --- \| --- \| \| OTU1 \| 4.67E-06 \| 4.08E-05 \| 3.91E-05 \| 1.23E-06 \| \| OTU5 \| 0.005898 \| 0.051067 \| 0.009938 \| 2.50E-05 \| \| OTU14 \| 0.005898 \| 0.027935 \| 0.006551 \| 7.18E-06 \| \| OTU20 \| 0.629161 \| 0.015568 \| 0.004028 \| 0.009873 \|   **Supplementary Table** **5.** FDR-corrected p-value of differences in relative abundance of a given OTU between different time points   \| **Time** \| **Ethanol** \| \| \| \| \| --- \| --- \| --- \| --- \| --- \| \| **OTU_1** \| **OTU_14** \| **OTU_5** \| **OTU_20** \| \| 3-10d - 4m \| 5.61E-01 \| 9.56E-01 \| 6.31E-01 \| 9.56E-01 \| \| 4m - 1y \| 1.73E-05 \| 5.79E-06 \| 3.10E-06 \| 6.48E-02 \| \| 1y - 2y \| 6.91E-01 \| 6.76E-01 \| 6.91E-01 \| 6.76E-01 \| \| **Time** \| **Ethanol + EMA** \| \| \| \| \| **OTU_1** \| **OTU_14** \| **OTU_5** \| **OTU_20** \| \| 3-10d - 4m \| 9.44E-01 \| 9.44E-01 \| 9.44E-01 \| 9.44E-01 \| \| 4m - 1y \| 4.26E-06 \| 9.44E-04 \| 7.36E-03 \| 1.62E-01 \| \| 1y - 2y \| 6.21E-01 \| 6.21E-01 \| 2.29E-01 \| 5.78E-01 \|   **Supplementary Table 6**. Persistence of ethanol-EMA enriched OTUs in ethanol-EMA treated samples from children | | | | | | |
| --- | --- | --- | --- | --- | --- | --- | --- | --- | --- | --- | --- | --- | --- | --- | --- | --- | --- | --- | --- | --- | --- | --- | --- | --- | --- | --- | --- | --- | --- | --- | --- | --- | --- | --- | --- | --- | --- | --- | --- | --- | --- | --- | --- | --- | --- | --- | --- | --- | --- | --- | --- | --- | --- | --- | --- | --- | --- | --- | --- | --- | --- | --- | --- | --- | --- | --- | --- | --- | --- | --- | --- | --- | --- | --- | --- | --- | --- | --- | --- | --- | --- | --- | --- | --- | --- | --- | --- | --- | --- |
| OTU number | Class | Number of children in who OTU was detected at | | | | |
|  |  | 10 days | All subsequent samplings | Two subsequent samplings | One subsequent sampling | Only 10 days |
| 1 | Clostridia | 26 | 20 | 5 | 1 | 0 |
| 14 | Clostridia | 18 | 11 | 7 | 0 | 0 |
| 5 | Clostridia | 20 | 14 | 5 | 1 | 0 |
| 20 | Bacilli | 15 | 3 | 10 | 2 | 0 |

| **Supplementary Table 7**. Number of colonies picked for 16S rRNA gene sequencing. By default, 10 or 12 colonies per sample were picked, if less than 10 colonies were grown, all of them were used for further DNA extraction and sequencing | | | | | | | | |
| --- | --- | --- | --- | --- | --- | --- | --- | --- |
| SampleID | Anaerobic | | | | Aerobic | | | |
|  | Mother | Child | | | Mother | Child | | |
|  |  | 3/10 days | 4 months | 1/2 years |  | 3/10 days | 4 months | 1/2 years |
| S1 | 10 | 10d: 10 | NG* | 1y: 19 | NG* | NG* | NG* | NG* |
| S2 | 10 | 10d: NG* | 10 | 1y: 10; 2 y: 5 | NG* | NG* | NG* | NG* |
| S3 | 10 | 10d: NG* | 12 | 1y: 6; 2y: 10 | NG* | NG* | NG* | NG* |
| S4 | 3 | 3d: 12 | 3 | 1y: 1 | NG* | 3d: 7 | NG* | NG* |
| S5 | 10 | 3d: 12 | 10 | 1y: 10 | NG* | NG* | NG* | 1y: 3 |
| S6 | 10 | 3d: 12 | NG* | 1y: 3 | NG* | NG* | NG* | NG* |
| S7 | 10 | 10d: NG* | 2 | 1y: 10 | NG* | NG* | NG* | NG* |
| S8 | 9 | 3d: NG* | 10 | 1y: 10 | NG* | NG* | NG* | NG* |
| Total | 249 | | | | 10 | | | |
| *NG: no growth | |  |  |  |  |  |  |  |

**Supplementary Table 8**. Comparison between cultured isolates and Illumina sequencing data

| Mother-child pair | Sample | IsolatedSpecies | | | | | Number of isolates | | Number of Illumina reads | | |
| --- | --- | --- | --- | --- | --- | --- | --- | --- | --- | --- | --- |
|  |  |  |  |  |  |  |  |  | EMA | Ethanol | |
| S1 | Mother | *Clostridium celatum* | | | | | 1 | | 3474 | 170 | |
|  |  | *Clostridium disporicum* | | | | | 3 | | 3489 | 170 | |
|  |  | *Turicibacter sanguinis* | | | | | 2 | | 0 | 1 | |
|  |  | *[Clostridium] dakarense* | | | | | 1 | | 9 | 1 | |
|  | 10 days | *Intestinibacter bartlettii* | | | | | 2 | | 0 | 0 | |
|  | 1 year | *Flavonifractor plautii* | | | | | 1 | | 0 | 0 | |
|  |  | *Intestinibacter bartlettii* | | | | | 2 | | 0 | 0 | |
|  |  | *Peptoclostridium difficile* | | | | | 9 | | 173 | 337 | |
|  |  | *Paeniclostridium sordellii* | | | | | 1 | | 0 | 0 | |
| S2 | Mother | *Peptostreptococcus russellii* | | | | | 1 | | 0 | 0 | |
|  | 4 months | *Clostridium perfringens* | | | | | 3 | | 24 | 109 | |
|  | 2 years | *Intestinibacter bartlettii* | | | | | 2 | | 236 | 88 | |
|  |  | *[Clostridium] saccharogumia* | | | | | 1 | | 0 | 0 | |
| S3 | Mother | *Clostridium chauvoei* | | | | | 1 | | 1573 | 405 | |
|  |  | *Turicibacter sanguinis* | | | | | 1 | | 0 | 2 | |
|  | 4 months | *Intestinibacter bartlettii* | | | | | 2 | | 623 | 81 | |
|  |  | *Paeniclostridium sordellii* | | | | | 7 | | 1124 | 145 | |
|  | 2 years | *Faecalicatena fissicatena* | | | | | 1 | | 0 | 0 | |
|  |  | *Flavonifractor plautii* | | | | | 1 | | 0 | 0 | |
|  |  | *Turicibacter sanguinis* | | | | | 3 | | 0 | 0 | |
|  |  | *[Clostridium] dakarense* | | | | | 3 | | 0 | 0 | |
|  |  | *[Clostridium] saccharogumia* | | | | | 2 | | 0 | 0 | |
|  | 1 year | *Blautia luti BInIX* | | | | | 4 | | 8 | 746 | |
|  |  | *[Clostridium] bolteae JCM 12243* | | | | | 2 | | 0 | 0 | |
| S4 | Mother | *Sellimonas intestinalis* | | | | | 3 | | 1 | 504 | |
|  | 3 days | *Bacillus circulans** | | | | | 4 | | 0 | 0 | |
|  |  | *Robinsoniella peoriensis* | | | | | 1 | | 0 | 0 | |
|  |  | *Ruminococcus gnavus* | | | | | 3 | | 0 | 0 | |
|  |  | *[Clostridium] dakarense* | | | | | 2 | | 0 | 0 | |
|  | 4 months | *Sellimonas intestinalis* | | | | | 3 | | 13 | 11 | |
|  | 1 year | *Erysipelatoclostridium ramosum* | | | | | 1 | | 0 | 0 | |
| S5 | Mother | *Clostridium celatum* | | | | | 1 | | 0 | 0 | |
|  |  | *Clostridium disporicum* | | | | | 3 | | 17 | 6 | |
|  | 3 days | *Clostridium perfringens* | | | | | 6 | | 0 | 0 | |
|  | 4 months | *Erysipelatoclostridium ramosum* | | | | | 8 | | 455 | 12178 | |
|  |  | *Flavonifractor plautii* | | | | | 1 | | 0 | 0 | |
|  | 1 year | *Bacillus thuringiensis** | | | | | 3 | | 0 | 0 | |
|  |  | *Clostridium perfringens* | | | | | 1 | | 0 | 0 | |
|  |  | *Flavonifractor plautii* | | | | | 1 | | 0 | 0 | |
|  |  | *[Clostridium] bolteae* | | | | | 1 | | 0 | 0 | |
|  |  | *[Clostridium] symbiosum* | | | | | 1 | | 0 | 0 | |
| S6 | Mother | *Blautia obeum* | | | | | 1 | | 0 | 35 | |
|  |  | *Clostridium disporicum* | | | | | 4 | | 91 | 145 | |
|  | 3 days | *Clostridium celatum* | | | | | 1 | | 0 | 0 | |
|  |  | *Clostridium disporicum* | | | | | 10 | | 0 | 0 | |
|  | 1 year | *Erysipelatoclostridium ramosum* | | | | | 1 | | 0 | 0 | |
|  |  | *Hungatella hathewayi* | | | | | 1 | | 0 | 0 | |
| S7 | Mother | *Blautia obeum* | | | | | 1 | | 29 | 388 | |
|  |  | *Clostridium celatum* | | | | | 3 | | 649 | 18 | |
|  |  | *Clostridium chromiireducens* | | | | | 1 | | 0 | 0 | |
|  |  | *Clostridium disporicum* | | | | | 1 | | 280 | 7 | |
|  |  | *Clostridium sartagoforme* | | | | | 2 | | 326 | 9 | |
|  |  | *[Eubacterium] rectale* | | | | | 2 | | 1816 | 1634 | |
|  | 4 months | *UNVERIFIED: [Clostridium]* | | | | | 1 | | 25 | 2 | |
|  |  | *[Clostridium] citroniae* | | | | | 1 | | 22 | 2 | |
|  | 1 year | *Clostridium uliginosum* | | | | | 1 | | 0 | 0 | |
|  |  | *Intestinimonas butyriciproducens* | | | | | 2 | | 0 | 0 | |
|  |  | *[Clostridium] citroniae* | | | | | 1 | | 0 | 0 | |
|  |  | *[Clostridium] symbiosum* | | | | | 1 | | 0 | 0 | |
| S8 | Mother | *Blautia obeum* | | | | | 1 | | 2 | 279 | |
|  |  | *Ruminococcus lactaris* | | | | | 6 | | 0 | 423 | |
|  | 4 months | *Sellimonas intestinalis* | | | | | 2 | | 26 | 24 | |
|  |  | *[Clostridium] innocuum* | | | | | 5 | | 1 | 32 | |
|  | 1 year | *Intestinibacter bartlettii* | | | | | 1 | | 0 | 0 | |
|  |  | *Paeniclostridium sordellii* | | | | | 6 | | 0 | 0 | |
|  |  | *Ruminococcus gnavus* | | | | | 3 | | 0 | 0 | |
| **Supplementary Table 9**. Similarity between cultured isolates and ethanol-EMA enriched OTUs | | | | | | | | | | |  |
| Isolate species assignment | | | Number of isolates | Closest OTU | Length | Identity | | E-value | | |  |
|  |  |  |  |  | Towards closest OTU | | | | | |  |
| *[Clostridium] dakarense* | | | 6 | OTU_1 | 314 | 97.6 | | 3.33E-89 | | |  |
| *Clostridium celatum* | | | 3 | OTU_14 | 364 | 98.3 | | 8.3E-163 | | |  |
| *Clostridium disporicum* | | | 21 |  | 362 | 98.2 | | 2E-154 | | |  |
| *Clostridium sartagoforme* | | | 2 |  | 304 | 99.8 | | 2E-160 | | |  |
| *Turicibacter sanguinis* | | | 5 | OTU_20 | 304 | 99.5 | | 5.3E-158 | | |  |
| *Intestinibacter bartlettii* | | | 7 | OTU_5 | 263 | 98.2 | | 1.67E-70 | | |  |
| *Paeniclostridium sordellii* | | | 9 |  | 303 | 98.68 | | 2E-154 | | |  |

| **Supplementary Table 10**. Best matching mother-child pairwise comparison between isolates of the same species | | | | |
| --- | --- | --- | --- | --- |
| Mother-child ID | Child age | Isolate | Pairwise identity (%) | Length, bp |
|  |  |  |  |  |
| S3 | 2 years | *Turicibacter sanguinis* | 98.8 | 880 |
| S4 | 4 months | *Sellimonas intestinalis* | 99.7 | 771 |
| S6 | 3 days | *Clostridium disporicum* | 99.7 | 1031 |
